# Supplementary material for: Diminishment of Nrf2 Antioxidative Defense Aggravates Nephrotoxicity of Melamine and Oxalate Coexposure
Source: Antioxidants (Basel). 2021 Sep 14;10(9):1464. doi: 10.3390/antiox10091464 (PMC8471505; doi:10.3390/antiox10091464)
Supplement: Supplementary file 1 [file antioxidants-10-01464-s001.zip › antioxidants-1345179-supplementary.pdf]

Supplementary Materials

Diminishment of Nrf2 antioxidative defense aggravates nephrotoxicity of melamine and oxalate coexposure

Chia-Fang Wu[1,2,+], Chia-Chu Liu[1,3-5,+], Yi-Chun Tsai[1,5,6], Chu-Chih Chen[1,7], Ming-Tsang Wu[1,8-10] and Tusty-Jiuan Hsieh [1,11,12,*]

[1] Research Center for Environmental Medicine, Kaohsiung Medical University, Kaohsiung, Taiwan

[2] International Master Program of Translational Medicine, National United University, Miaoli, Taiwan

[3] School of Medicine, College of Medicine, Kaohsiung Medical University, Kaohsiung, Taiwan

[4] Department of Urology, Kaohsiung Medical University Hospital, Kaohsiung Medical University, Kaohsiung, Taiwan

[5] Department of Urology, Pingtung Hospital, Ministry of Health and Welfare, Pingtung City, Taiwan

[6] Divisions of Nephrology, Kaohsiung Medical University Hospital, Kaohsiung Medical University, Kaohsiung, Taiwan

[7] Institute of Population Health Sciences, National Health Research Institutes, Miaoli, Taiwan

[8] Ph.D. Program of Environmental and Occupational Medicine, College of Medicine, Kaohsiung Medical University, Kaohsiung, Taiwan

[9] Graduate Institute of Clinical Medicine, College of Medicine, Kaohsiung Medical University, Kaohsiung, Taiwan

[10] Department of Family Medicine, Kaohsiung Medical University Hospital, Kaohsiung Medical University, Kaohsiung, Taiwan

[11] Graduate Institute of Medicine, College of Medicine, Kaohsiung Medical University, Kaohsiung, Taiwan

[12] Department of Marine Biotechnology and Resources, College of Marine Sciences, National Sun Yat-Sen University, Kaohsiung, Taiwan

[+]Both equal contribution

* Corresponding author: Tusty-Jiuan Hsieh, Ph.D.

Address: No. 100 Shih-Chuan 1st Rd, Graduate Institute of Medicine, College of Medicine, Kaohsiung Medical University, Kaohsiung 807, Taiwan

E-mail: hsiehjun@kmu.edu.tw

Telephone: +886 7 3121101, ext. 2759#423

ORCID: 0000-0001-5387-6522

Supplementary Figure

(A)

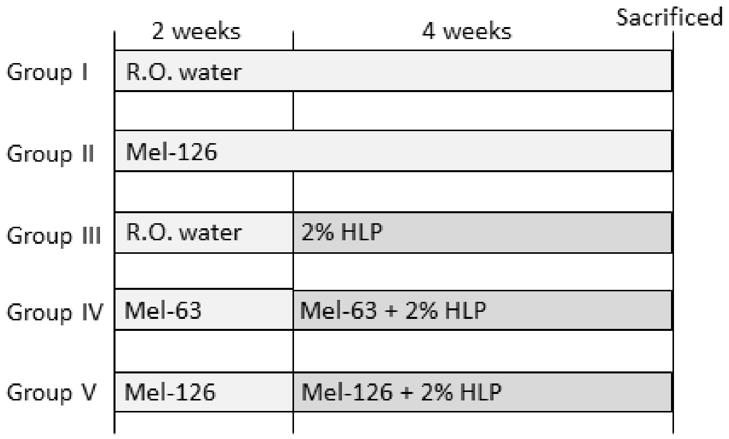

(B)

	Animal numbers		
	Batch 1	Batch 2	Batch 3
Group I	3	4	6
Group II	0	3	6
Group III	3	4	6
Group IV	3	4	6
Group V	3	4	6

Figure S1. Scheme of the animal study. (A) Experimental design of the animal study. The Sprague-Dawley male rats were randomly distributed into five groups: Group I (Control); Group II (126 mg/kg/day melamine); Group III (2% HLP); Group IV (63 mg/kg/day melamine + 2% HLP); and Group V (126 mg/kg/day melamine + 2% HLP). For Group II, IV and V, the rats were pre-exposed to melamine in drinking water for 2 weeks and then co-exposed to melamine and HLP in drinking water for another 4 weeks. (B) Animal numbers in each group and batch of the animal study.

Supplementary Tables

Table S1. Analysis of urinary sediments in the rats

First batch experiment

Group	I: Control (n = 3)			II: M-126 (n = 0)	III: HLP (n = 3)			IV: M-63+HLP (n = 3)			V: M-126+HLP (n = 3)		
Rat Id.	1	2	3		4	5	6	7	8	9	10	11	12
CO	–	–	1+		–	–	1+	–	1+	2+	2+	2+	3+
AR (AP)	–	1+	–		–	–	–	1+	–	–	–	–	–
TP	–	–	–		2+	2+	–	1+	–	–	–	–	–
Incidence of CO in urine		1/3				1/3			2/3			3/3	

Second batch experiment

Group	I: Control (n = 4)				II: M-126 (n = 3)			III: HLP (n = 4)				IV: M-63+HLP (n = 4)				V: M-126+HLP (n = 4)			
Rat Id.	2	3	4	5	7	8	9	11	12	13	14	16	17	18	19	21	22	23	24
CO	–	–	–	–	1+	–	–	–	–	–	–	2+	2+	–	1+	2+	3+	1+	–
AR (AP)	–	–	–	–	–	–	–	–	–	–	–	–	–	–	–	–	–	–	–
TP	2+	1+	1+	–	–	–	–	1+	1+	1+	–	–	–	1+	1+	–	–	1+	+/–
Incidence of CO in urine	0/4				1/3			0/4				3/4				3/4			
Total incidence of CO in urine	1/7				1/3			1/7				5/7				6/7			

Mel-126: 126 mg/kg/day melamine; HLP: 2% hydroxy-L-proline; Mel-63+HLP: 63 mg/kg/day melamine + 2% hydroxy-L-proline; Mel-126+HLP: 126 mg/kg/day melamine + 2% hydroxy-L-proline; Rat Id: identification of rats.
CO: Calcium oxalate; AP: Amorphous phosphate; AR: Amorphous urate; TP: Triple phosphate.
The result was analyzed by an experience medical laboratory technologist who was blinded to the study design.
The frequency of sediments presented in 10 fields under a microscope (100×): 25% = 1+; 50% = 2+; 75% = 3+
Note: The rats that assigned Id. 1, 6, 10, 15 and 20 were removed from this study for the other research purpose.

Table S2. Purchase information for antibodies

Antibody	Catalog No.	Supplier
Cluster of differentiation 68 (CD68, ED-1)	ab31630	Abcam Plc., Cambridge, MA, USA
Collagen IV	ab6586	
Transforming growth factor beta 1 (TGF-β1)	ab92486	
Kidney injury molecule 1 (KIM-1)	OACD04979	Aviva Systems Biology Corporation, San Diego, CA, USA
Caspase-3	#9662	Cell Signaling Technology, Danvers, MA, USA
Cleaved-caspase-3 (Asp175)	#9661	
NAD(P)H dehydrogenase quinone 1 (NQO1)	#62262	
Nuclear factor-kappa B (NF-κB) p65	#8242	
Phospho-NF-κB p65	#3033	
Heme oxygenase 1 (HO1)	ADI-SPA-895	Enzo Life Sciences, Inc., Farmingdale, NY, USA
8-oxoguanine glycosylase 1 (OGG1)	GTX20204	GeneTex, Inc., Irvine, CA, USA
Beta actin	GTX109639	
Malondialdehyde (MDA)	GTX12835	
Superoxide dismutase 1 (SOD1)	GTX100659	
Superoxide dismutase 2 (SOD2)	GTX116093	
8-oxo-2′-deoxyguanosine (8-OHdG)	N45.1	JalCA, Fukuroi, Shizuoka, Japan
Nuclear factor (erythroid-derived 2)-like 2 (Nrf2)	NBP1-32822	Novus Biologicals LCC., Centennial, CO, USA
Kelch-like ECH-associated protein 1 (Keap1)	MAB3024	R&D Systems, Inc., Minneapolis, MN, USA
Catalase	sc-50508	Santa Cruz Biotechnology, Inc., Dallas, TX, USA
Histone 3	sc-10809	

Table S3. Clinical biochemical parameters from the rats

	Control (n=10)		Mel-126 (n=9)		HLP (n=10)		Mel-63+HLP (n=10)		Mel-126+HLP (n=10)		
	Mean	SE	Mean	SE	Mean	SE	Mean	SE	Mean	SE	p value
Body weight (g)	495.1	14.49	477.6	9.89	478.3	11.43	495.2	11.73	481.1	8.43	0.6567
Water intake (ml)	51.23	0.72	46.31	1.73	51.15	0.20	52.14	0.22	47.12	1.14	<0.001
Urine amount (ml)	16.55	0.84	16.83	1.12	20.40	1.14	26.90	2.98	25.30	2.80	0.0014
Serum CREA (mg/dl)	0.23	0.02	0.25	0.01	0.24	0.01	0.24	0.01	0.24	0.02	0.9016
Serum BUN (mg/dl)	14.87	0.78	16.55	0.82	13.84	0.73	15.50	0.53	18.10	0.93	0.0033
Serum UA (mg/dl)	3.71	1.04	4.66	0.75	5.35	1.11	6.59	0.71	5.48	0.91	0.2728
Serum P (mg/dl)	8.56	0.34	9.78	0.65	11.87	0.96	11.41	0.21	10.30	0.49	0.0017
Serum Ca (mg/dl)	11.07	0.54	11.73	0.45	12.20	0.48	13.16	0.29	13.22	0.25	0.0021
Urine Ox (μmol/day)#	51.84	4.37	55.39	4.26	77.88	5.92	93.52	10.95	80.14	7.44	0.0012
Urine CREA (mg/day)	11.27	0.77	13.25	0.55	14.12	0.77	15.95	1.35	15.27	1.10	0.0118
Urine BUN (g/day)	0.47	0.04	0.50	0.03	0.53	0.03	0.64	0.07	0.62	0.05	0.0400
Urine NAG (μg/day)	7.56	1.60	5.70	1.08	20.44	4.89	17.79	4.68	14.90	1.78	0.0015
Urine β2M (μg/day)	1.45	0.45	2.15	0.77	0.71	0.10	1.79	0.44	0.73	0.10	0.0879

Mel-126: 126 mg/kg/day melamine; HLP: 2% hydroxy-L-proline; Mel-63+HLP: 63 mg/kg/day melamine + 2% hydroxy-L-proline; Mel-126+HLP: 126 mg/kg/day melamine + 2% hydroxy-L-proline; SE: standard error; CREA: creatinine; BUN: blood urea nitrogen; UA: uric acid; P: phosphorus; Ca: calcium; Ox: oxalate; NAG: N-acetyl-β-D-glucosaminidase; β2M: Beta-2-microglobulin.
Data combined from the second and the third batch animal experiments.
#Note: n=6 for each group (only measured in rats from the third batch animal experiment)

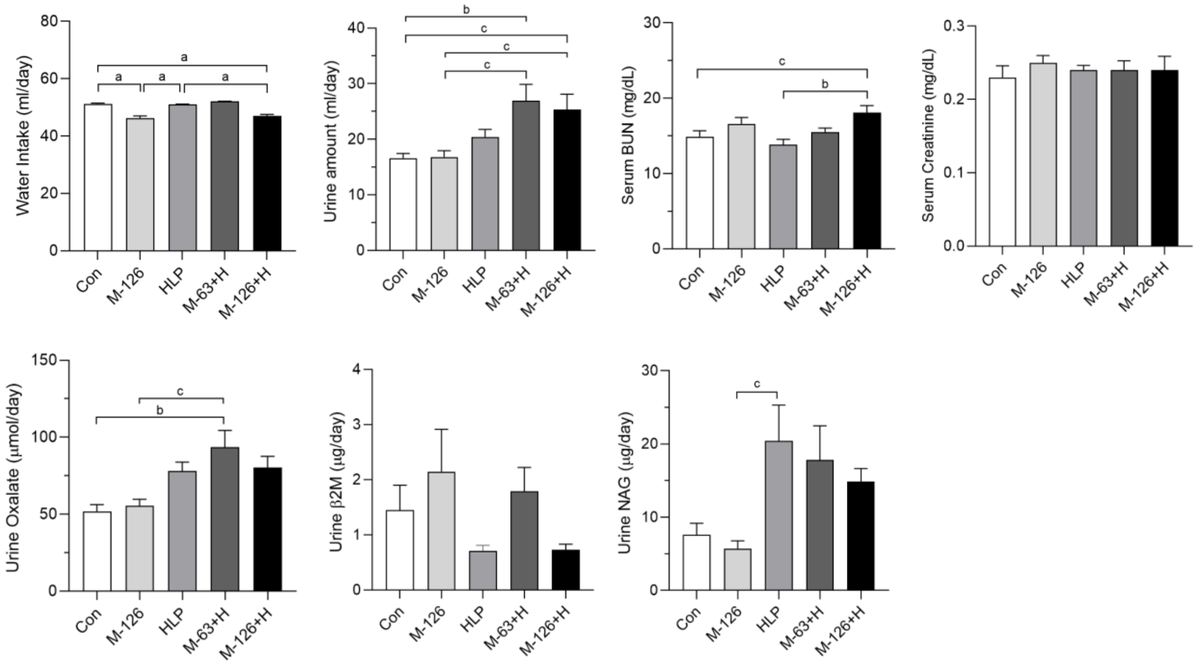

Supplementary Figure: The statistic results of between-group comparisons for the clinical biochemical parameters from Table S3.
Con: control; HLP or H: 2% hydroxy-L-proline; M-126: 126 mg/kg/day melamine; M-63: 63 mg/kg/day melamine. One-way analysis of variance (ANOVA) followed by Tukey's multiple comparison test was used to analyze the differences across groups. Data are mean ± SE. a: $p<0.05$; b: $p<0.01$; c: $p<0.001$.
